# P-2343. Incidence of Diagnosed Herpes Zoster and Risk of Complications by Race and Ethnicity: A Retrospective Cohort Study Among Adults in the United States

**DOI:** 10.1093/ofid/ofae631.2495

**Published:** 2025-01-29

**Authors:** Nikita Stempniewicz, Andrea Steffens, Yong Zhu, Mary DuCharme, Stephanie Gallagher, Jessica Pickett, Justin Gatwood

**Affiliations:** GSK, Philadelphia, Pennsylvania; OPTUM, Eden Prairie, Minnesota; OPTUM, Eden Prairie, Minnesota; OPTUM, Eden Prairie, Minnesota; OPTUM, Eden Prairie, Minnesota; GSK, Philadelphia, Pennsylvania; GSK, Philadelphia, Pennsylvania

## Abstract

**Background:**

Herpes zoster (HZ) is characterized by a painful dermatomal rash. Recent US estimates for HZ incidence and risk of HZ complications by race and ethnicity are limited. This study aimed to estimate the incidence of diagnosed HZ, and among HZ cases, the proportion of HZ-related complications and hospitalizations by race and ethnicity.

**Figure:**
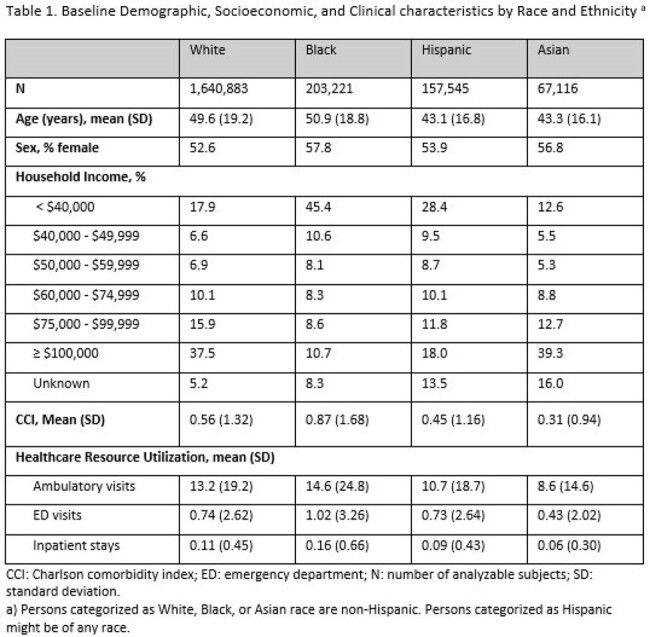

**Methods:**

Adults (aged ≥ 18 years) identified from linked US electronic health record (EHR) and administrative claims data (2015-2023) for Medicare Advantage with Part D and commercial insurance enrolees, were assigned to four cohorts based on race and ethnicity in the EHR: White, Black, Hispanic, and Asian. Index dates were randomly assigned after 12 months of baseline continuous enrolment, and individuals were followed until the earlier of an incident HZ diagnosis or censoring event (HZ vaccination, disenrollment, death, or end of the study period). Individuals with a baseline HZ diagnosis or HZ vaccination were excluded. Outcomes included the incidence of diagnosed HZ, and among HZ cases, the proportion with HZ-related complications and hospitalizations. Outcomes were described in each cohort and compared using multivariable analysis adjusting for baseline covariates.

**Figure:**
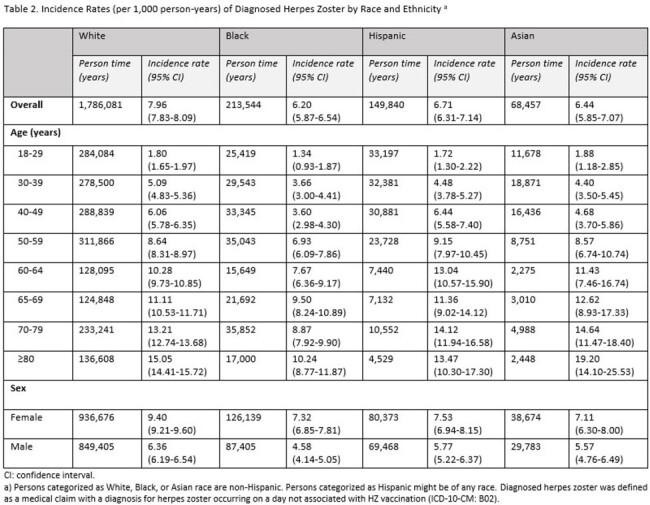

**Results:**

Overall, 1,640,883 White, 203,221 Black, 157,545 Hispanic, and 67,116 Asian individuals were included in the analytical sample (Table 1). HZ incidence per 1,000 person-years for these cohorts was 7.96, 6.20, 6.71, and 6.44, respectively (Table 2). Among diagnosed HZ cases, the proportion with postherpetic neuralgia (PHN) and HZ-related hospitalizations was numerically highest in the Black cohort (Table 3). After multivariable analysis, HZ incidence rates were lower and the odds of PHN and HZ-related hospitalization were higher among Black compared to White individuals (p < 0.01, Table 4). No significant differences in adjusted outcomes were observed between the Asian and Hispanic cohorts and White cohort.

**Figure:**
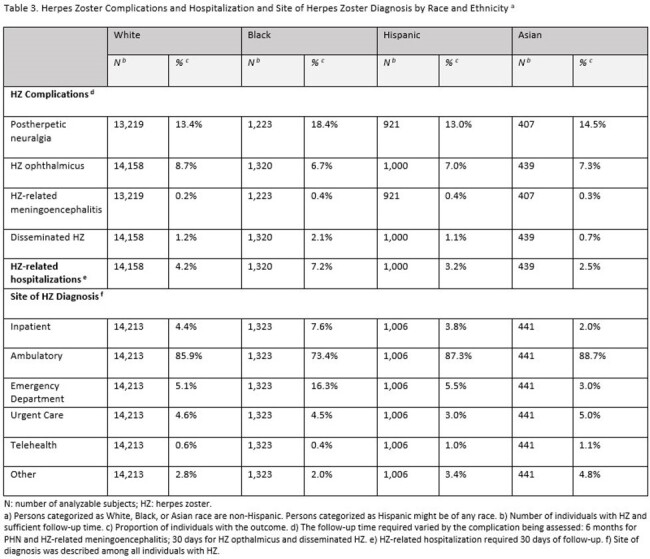

**Conclusion:**

In this insured population, the incidence of diagnosed HZ was lower but the odds of PHN and HZ-related hospitalization were higher among Black compared to White adults. Further research is needed to understand reasons for these differences, including the potential underdiagnosis of HZ in certain populations or sites of care.

FUNDING: GSK (VEO-000678)

**Figure:**
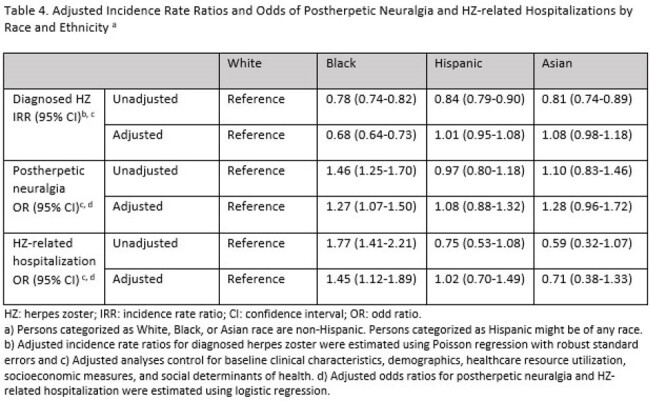

**Disclosures:**

Nikita Stempniewicz, MSc, GSK: Employment|GSK: Stocks/Bonds (Public Company) Andrea Steffens, MPH, OPTUM: Employee|UnitedHealth Group: Stocks/Bonds (Public Company) Yong Zhu, PhD, GSK: Funding through OPTUM|OPTUM: Employee Mary DuCharme, MLIS, GSK: Funding through OPTUM|OPTUM: Employee Stephanie Gallagher, MPH, GSK: Funding through OPTUM|OPTUM: Employee Jessica Pickett, PhD, GSK: Employee Justin Gatwood, PhD, MPH, AstraZeneca: Grant/Research Support|Genentech: Advisor/Consultant|GSK: Employment|GSK: Stocks/Bonds (Public Company)|Merck & Co.: Advisor/Consultant|Merck & Co.: Grant/Research Support

